# Improvement of cardiomyocyte function by in vivo hexarelin treatment in streptozotocin‐induced diabetic rats

**DOI:** 10.14814/phy2.13612

**Published:** 2018-02-14

**Authors:** Xinli Zhang, Linbing Qu, Ling Chen, Chen Chen

**Affiliations:** ^1^ School of Biomedical Sciences University of Queensland St Lucia Brisbane Queensland Australia; ^2^ State Key Laboratories of Respiratory Diseases Guangzhou Institutes of Biomedicine and Health Chinese Academy of Sciences Guangzhou China

**Keywords:** Apoptosis, calcium, cardiomyocytes, diabetes, hexarelin

## Abstract

Diabetic cardiomyopathy is characterized by diastolic and systolic cardiac dysfunction, yet no therapeutic drug to specifically treat it. Hexarelin has been demonstrated to improve heart function in various types of cardiomyopathy via its receptor GHS‐R. This experiment aims to test the effect of hexarelin on cardiomyocytes under experimental diabetes. Streptozotocin (STZ, 65 mg/kg)‐induced diabetic rat model was employed with vehicle injection group as control. Daily hexarelin (100 *μ*g/kg) treatment was performed for 2 weeks after 4‐week STZ‐induced diabetes. Cardiomyocytes were isolated by enzyme treatment under O^2^‐saturated perfusion for single‐cell shortening, [Ca^2+^]_i_ transient, and electrophysiology recordings. GHS‐R expression and apoptosis‐related signaling proteins Bax, Bcl‐2, caspase‐3 and 9, were assessed by western blot. Experimental data demonstrated a reduced cell contraction and relaxation in parallel with depressed rise and fall of [Ca^2+^]_i_ transients in diabetic cardiomyocytes. Hexarelin reversed the changes in both contraction and [Ca^2+^]_i_. Action potential duration and transient outward potassium current (*I*_to_) density were dramatically increased in diabetic cardiomyocytes and hexarelin treatment reverse such changes. Upregulated GHS receptor (GHS‐R) expression was observed in both control and diabetic groups after hexarelin treatment, which also caused antiapoptotic changes of Bax, Bcl‐2, caspase‐3 and 9 expression. In STZ‐induced diabetic rats, hexarelin is able to improve cardiomyocyte function through recovery of *I*_to_ K^+^ currents, intracellular Ca^2+^ homeostasis and antiapoptotic signaling pathways.

## Introduction

Diabetic cardiomyopathy (DCM) has been recognized for decades after Rubler first found this clinical entity without the presence of hypertension or coronary artery diseases in 1972 (Rubler et al. 1972). It is characterized by cardiac dysfunction initiated by significantly diastolic disorders. Indeed, clinical evidence suggested that diabetic patients had obvious diastolic abnormalities but not significant systolic dysfunction in earlier phase of DCM (Hayat et al. 2004). Echocardiography studies also showed that systolic dysfunction often occurred long after establishment of diastolic dysfunction (Petrie et al. 2002).

STZ‐induced diabetic animal model has been used to investigate DCM in many studies (Teshima et al. 2000; Choi et al. 2002; Rithalia et al. 2004; Singh et al. 2006; Aragno et al. 2012). Defect in dynamic changes of intracellular calcium ([Ca^2+^]i) occurred causing reduced cardiac contraction force and relaxation velocity (Choi et al. 2002; Rithalia et al. 2004). Action potential (AP) is a hallmark linking directly to myocardial contraction and relaxation, especially tightly linked at the single‐cardiomyocyte level. Previous studies on isolated cardiomyocytes from diabetic animals revealed that there was no change on AP amplitude but prolonged AP duration (APD) (Nobe et al. 1990; Shigematsu et al. 1994). In forming action potential, *I*_to_ K^+^ current is mainly responsible for APD among all transmembrane ion currents in cardiomyocyte (Casis et al. 1998; Greenstein et al. 2000). Experimental study on diabetic cardiomyocytes exhibited reduced *I*_to_ current causing an increase in APD with delayed AP repolarization and a prolonged duration of Ca^2+^ influx through voltage‐gated Ca^2+^ channels (Yu et al. 1997). It was also reported that prolonged APD inhibited Ca^2+^ efflux, causing cardiac diastolic dysfunction in DCM (Shigematsu et al. 1994). Cell death is another contributor to cardiac dysfunction or loss of cardiac contractility (Swynghedauw 1999). STZ‐induced diabetic rat study exhibited that Bcl‐2 expression was significantly decreased with elevated Bax protein content in diabetic cardiomyocytes (Westermann et al. 2007). Hyperglycemia‐induced myocardium showed that apoptosis was mediated by increased caspase‐3 activity (Cai et al. 2002). Inhibited caspase‐3 and caspase‐9 activation was effectively regulating the levels of proteins related to apoptosis in H9c2 cells exposed to high‐glucose (Sun et al. 2014).

Growth hormone secretagogues (GHSs), including ghrelin and its synthetic analogue hexarelin, have exhibited cardio‐protective effect in cardiomyopathy. Ghrelin, a 28 amino acid peptide produced in stomach, is the endogenous ligand of GHS receptor (GHS‐R) (Kojima et al. 1999). Synthetic GHS hexarelin also binds and activates GHS‐R (Bodart et al. 2002; Torsello et al. 2003; Bulgarelli et al. 2009). Previous studies in this laboratory have shown that ghrelin and hexarelin are able to recover contractility, transient [Ca^2+^]_i_ and ion channel activities in ischemia/reperfusion injured cardiomyocytes (Ma et al. 2012, 2012). Besides, hexarelin may attenuate cardiac fibrosis in hypertensive rats and protect cardiomyocytes from angiotensin‐II‐induced apoptosis (Pang et al. 2004; Xu et al. 2012). Thus, we aim to investigate whether hexarelin has protective effect on impaired contraction, [Ca^2+^]i handling, ion channel properties and apoptosis of cardiomyocytes in streptozotocin (STZ)‐induced diabetic rats.

## Materials and Methods

### Diabetic rat model

Male Wistar rats (6‐week‐old), weighing 150–200 g, were used in the study. Animals were distributed into 2 groups: 24 nontreated control (NC) and 24 nontreated diabetes (ND). Diabetic animals were prepared by the injection intraperitoneally with a single dose of freshly prepared STZ (65 mg/kg) and control animals only received the vehicle as previously described (Flarsheim et al. 1996). Fasting blood glucose level was determined by using Accu‐Chek glucometer (Roche, Indianapolis, IN) 72 h and 6 weeks after STZ injection. Animals were considered to be diabetic if there fasting blood glucose was at least 16.6 mmol/L. After 4‐week disease development, 12 control (hexarelin‐treated control, HC) and 12 diabetic (hexarelin‐treated diabetes, HD) rats were intraperitoneally injected with hexarelin (100 *μ*g/kg) every day for 2 weeks, others were injected with vehicle buffer. All animal experiments conformed to the Guide for the Care and Use of Laboratory Animals published by the Australian National Health and Medical Research Council and was approved by the Animal Ethics Committees of The University of Queensland.

### Ventricular cardiomyocyte preparation

Ventricular cardiomyocyte isolation has been described previously (Sun et al. 2010b). Briefly, rats were anesthetized with sodium pentobarbitone (40 mg/kg). Hearts were rapidly excised, placed in ice‐cold Ca^2^‐free Tyrode solution, cannulated, and then perfused retrogradely with Ca^2^‐free Tyrode solution via the aorta on a *Langendorf* perfusion apparatus until spontaneous contractions ceased (~5 min). Following this, cardiomyocytes were isolated from the left ventricle of each heart with Tyrode solution containing 100 *μ*mol/L CaCl_2_, 0.5 mg/mL collagenase Type II (Worthington, NJ, USA) and 0.15 mg/mL proteinase Type XXIV (Sigma, MO, USA). The Ca^2+^ level was gradually increased to 1.5 mmol/L in 30 min. Quiescent cardiomyocytes with a rod shape, sharp edges and clear striations were used in the investigation.

### Measurement of sarcomere shortening and intracellular Ca^2+^ transient

Sarcomere shortening was measured as previously described (Sun et al. 2010a). In brief, isolated cardiomyocytes were electrically stimulated at 0.5 Hz until contraction become uniform and 15–20 consecutive contractions were recorded. The percentage of sarcomere shortening, time‐to‐peak, time‐to‐90% relaxation, rate of shortening and relaxation were determined by IonWizard software (IonOptix Corporation, MA).

For examination of intracellular Ca^2+^ transient, 5 *μ*mol/L Fura‐2 AM (Invitrogen, CA, USA) was incubated with cardiomyocytes for 10 min at room temperature. With stimulated cardiomyocytes at 0.5 Hz, Fura‐2 fluorescence signals were recorded by an IonOptix Hyperswitch dual‐excitation light source (IonOptix Corporation, MA) at 340 and 380 nm and emitted light collected in a photomultiplier tube. [Ca^2+^]_i_ concentration was inferred from the ratio (R) of the intensity of the emitted fluorescence signals. Amplitude, time‐to‐peak, time‐to‐90% decay, rate of rise and fall of the derived [Ca^2+^]_i_ transient were determined by IonWizard software.

### Electrophysiological recordings

The whole‐cell patch recordings were previously described with some modifications (Sun et al. 2010b). In brief, cardiomyocytes were placed in a perfusion chamber (0.45 mL) mounted on the stage of an inverted microscope. After 10 min settle down, cardiomyocytes were perfused with Tyrode solution with 1.5 mmol/L CaCl_2_ at a flow rate of 2–3 mL/min. All recordings were obtained at room temperature (20°C–23°C).

The glass microelectrodes were pulled from borosilicate glass capillaries with inner filaments (Harvard Apparatus Led., Edenbridge, UK) by Sutter P‐87 microelectrodes puller (Sutter Equipment Co., Novato, CA). The resistance of the recording pipette filled with pipette solution was 2–4 MΩ. All recordings were performed, using the Axonpatch 200A amplifier (Axon Instruments, Foster City, CA). In our experiment, capacitance and series resistance compensation were optimized and 60–75% compensation was usually obtained. Recorded signals were low‐pass filtered at 1 kHz. Data acquisition and analysis were conducted with pClamp program (version 10.2, Axon Instruments).

AP was elicited by 1.5‐fold excitation threshold current pulses of 3 mesec in duration with 2 sec intervals between pulses. Once the recordings were stabilized, 10 successive APs were recorded and the average calculated for analysis. The following parameters were obtained: AP amplitude, resting membrane potential (RMP), and APD at 20, 50, and 90% repolarization (APD_20_, APD_50_, and APD_90_, respectively).

To record *I*_to_, a 50 msec prestep of −40 mV from the holding potential of −80 mV was applied to inactivate *I*_Na_. *I*_to_ was then evoked by the following test pulses from −30 to +60 mV in 10 mV increments for 200 msec. Steady‐state inactivation of *I*_to_ was measured after a 500 msec conditioning prepulse from a holding potential of −80 mV to potentials between −100 and 0 mV in steps of 10 mV, followed by a test pulse to +80 mV for 200 msec.

### Western blotting

Protein expression of the GHS receptor GHS‐R, Bcl‐2, Bax, caspase‐3 and caspase‐9 was examined by Western blot analysis. In brief, proteins were extracted from minced rat left ventricles in lysis buffer. Extracted proteins was denatured at 70°C (GHS‐R) or 95°C (Bcl‐2, Bax, caspase‐3 and caspase‐9) for 10 min in 2× sample buffer, separated on 10% SDS‐polyacrylamide gels and transferred to nitrocellulose membranes. After blocking, the membrane was incubated with polyclonal rabbit anti‐GHS‐R (1:500; Abcam, Cambridge, MA, USA), anti‐Bcl‐2 (1:500, Cell Signalling Technology, Danvers, MA, USA), anti‐Bax (1:1000, Cell Signalling Technology, Danvers, MA, USA), anti‐caspase‐3 (1:1000, Cell Signalling Technology, Danvers, MA, USA) and anti‐caspase‐9 (1:1000, Cell Signalling Technology, Danvers, MA, USA) primary antibodies overnight at 4°C before incubation with the corresponding secondary antibody (1:10,000) and detection with enhanced chemiluminescence (Pierce) according to the manufacturer's instructions. Rat *β*‐tublin (1:2000) was used as the internal control to allow semiquantitative densitometry analysis on scanned films, using ImageJ software (Fung et al. 2010).

### Solutions and chemicals

The Tyrode solution had the following composition (in mmol/L): 10 HEPES, 143 NaCl, 5.4 KCl, 0.5 MgCl_2_, 10 glucose, 20 taurine, and 1.5 CaCl_2_ (pH 7.4 adjusted with NaOH). The intracellular recording pipette solution for AP and *I*_to_ contained (mmol/L) 5 EGTA, 10 HEPES, 25 KCl, 5 MgATP, 1 MgCl_2_, and 125 potassium aspartate (pH 7.25 with KOH). The AP was recorded with Tyrode solution in the bath. After attainment of the whole‐cell configuration in Tyrode solution, *I*_to_ was isolated from other overlapping currents by the addition of Ca^2+^ channel blocker nifedipine (100 nmol/L) and delayed rectifier K^+^ channel blocker tetraethylammonium chloride (TEA‐Cl, 50 mmol/L) to the bath solution to eliminate Ca^2+^ and delayed rectifier K^+^ currents, respectively.

### Statistical analysis

All data were expressed as mean ± SEM One‐way ANOVA with Tukey post hoc test was carried out for multiple comparisons as appropriate. In all comparisons, the differences were considered to be statistically significant at a value of *P* < 0.05.

## Results

### General features of diabetic rats with hexarelin treatment

As shown in Table 1, increased blood glucose level, decreased body and heart weight were observed in nontreated diabetic (ND) rats. After hexarelin treatment, body and heart weight significantly elevated in both hexarelin‐treated control (HC) and hexarelin‐treated diabetic (HD) rats compared to nontreated control (NC) and ND rats respectively. In addition, blood glucose level of HD rats was partially recovered in comparison with ND rats however that of HC rats remained no change. There was no significant difference in ratio of heart weight and body weight among groups.

**Table 1:** General characteristics of untreated control (NC), hexarelin‐treated control (HC), untreated diabetes (ND), and hexarelin‐treated diabetes (HD) rat

	NC	HC	ND	HD
Body weight (g)	390.3 ± 22.3***	458.6 ± 59.8##	270.5 ± 23.3	317.1 ± 26.0*
Heart weight (g)	1.25 ± 0.19***	1.64 ± 0.09###	0.88 ± 0.10	1.12 ± 0.13*
Heart weight/body weight	0.0031 ± 0.0006	0.0036 ± 0.0003	0.0034 ± 0.0004	0.0034 ± 0.0005
Blood glucose (mmol/L)	6.40 ± 0.55***	5.76 ± 0.75	29.40 ± 2.68	22.82 ± 4.32*

Data from NC, HC, ND and HD groups are shown in this Table with *n* = 16. Data are mean ± SEM. Statistical comparisons were performed with One‐way ANOVA analysis. Note that ^#^indicates comparison with NC group and *indicates comparison with ND group, ^##^ *P* < 0.01, ^###^ *P < *0.005, *P < 0.05, ***P < 0.001.

### Effect of hexarelin on sarcomere shortening and intracellular Ca^2+^ transients from control and diabetic rat cardiomyocytes

**Figure 1:**
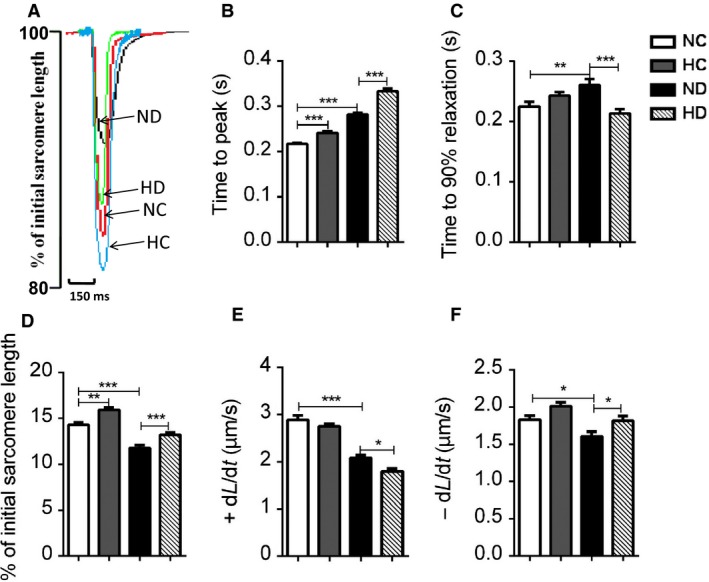
Effect of hexarelin on cardiomyocyte contractility from NC, HC, ND, and HD rats. (A) The representative superimposed traces of sarcomere shortening from 100% in different groups. Increased (D) amplitude of contraction (percentage sarcomere shortening used in this bar graph) was observed in both HC and HD group. Prolonged (C) time to 90% relaxation and (F) rate of relaxation in ND rats were normalized by hexarelin treatment, whereas further increased (B) time to peak and decreased (E) rate of contraction were observed in HD group. *n* = 81, 84, 90 and 87 cells/6 rats from NC, HC, ND and HD group, respectively. Data were shown as means ± SEM, *P < 0.05, **P < 0.01, ***P < 0.001.

**Figure 2:**
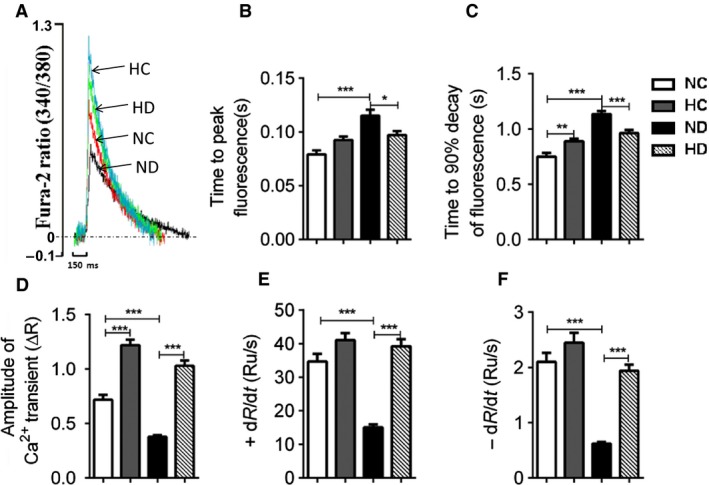
Effect of hexarelin on cardiomyocyte [Ca^2+^]_i_ transients from NC, HC, ND, and HD rats. The emission fluorescence ratio of Fura‐2 from excitation of 340 and 380 nm was short for R in the figure and Ru represented ratio unit. (A) The representative superimposed traces of cardiomyocyte [Ca^2+^]_i_ transient from different groups. Significantly increased (D) amplitude of contraction was observed in both HC and HD group. Prolonged (B) time to peak and (C) time to 90% decay of fluorescence in ND rats were normalized by hexarelin treatment. Hexarelin also recovered and further increased the rate of [Ca^2+^]_i_ transient (E) rise and (F) decay in HD and HC group. *n* = 63, 64, 68 and 65 cells/6 rats from NC, HC, ND and HD group, respectively. Data were shown as means ± SEM, *P < 0.05, **P < 0.01, ***P < 0.001.

The effects of hexarelin on control and diabetic rats cardiomyocytes function were determined by cell shortening and [Ca^2+^]_i_ transients (Figs. 1 and 2). All measurements were recorded with stimulation at 0.5 Hz.

Sarcomere shortening was expressed as percentage of the rest sarcomere length (Fig. 1A). It was found that relative sarcomere shortening was dramatically decreased in ND group compared to NC cardiomyocytes (Fig. 1D). Associated with slow rate of shortening and relaxation (Fig. 1E and F), ND rats also showed significantly increased time‐to‐peak and time‐to‐90% relaxation (Fig. 1B and C). After hexarelin treatment, cardiomyocytes of HD rats exhibited recovered relative sarcomere shortening, time‐to‐90% relaxation and rate of relaxation. Time‐to‐peak and rate of shortening, however, were prolonged compared to ND group. Increased relative sarcomere shortening and time course were also observed in HC cardiomyocytes (Fig. 1).

[Ca^2+^]_i_ transients were shown as cytoplasmic fura‐2 ratio changes. Accompanied with decreased sarcomere shortening, a reduction in amplitude of [Ca^2+^]_i_ transients was observed in ND rats compared to NC group (Fig. 2D). In addition, ND cardiomyocytes showed slow time‐to‐peak and time‐to‐90% decay (Fig. 2B and C), reduced rate of [Ca^2+^]_i_ transients development and decline in comparison with NC rats (Fig. 2E and F). Hexarelin treatment recovered impaired [Ca^2+^]_i_ transients amplitude, prolonged the time courses and rate of rise and fall in STZ‐treated rats. Besides, hexarelin elevated all measured parameters of [Ca^2+^]_i_ transients in control group.

### Effect of hexarelin on AP and *I*_to_ from control and diabetic rat cardiomyocytes

**Figure 3:**
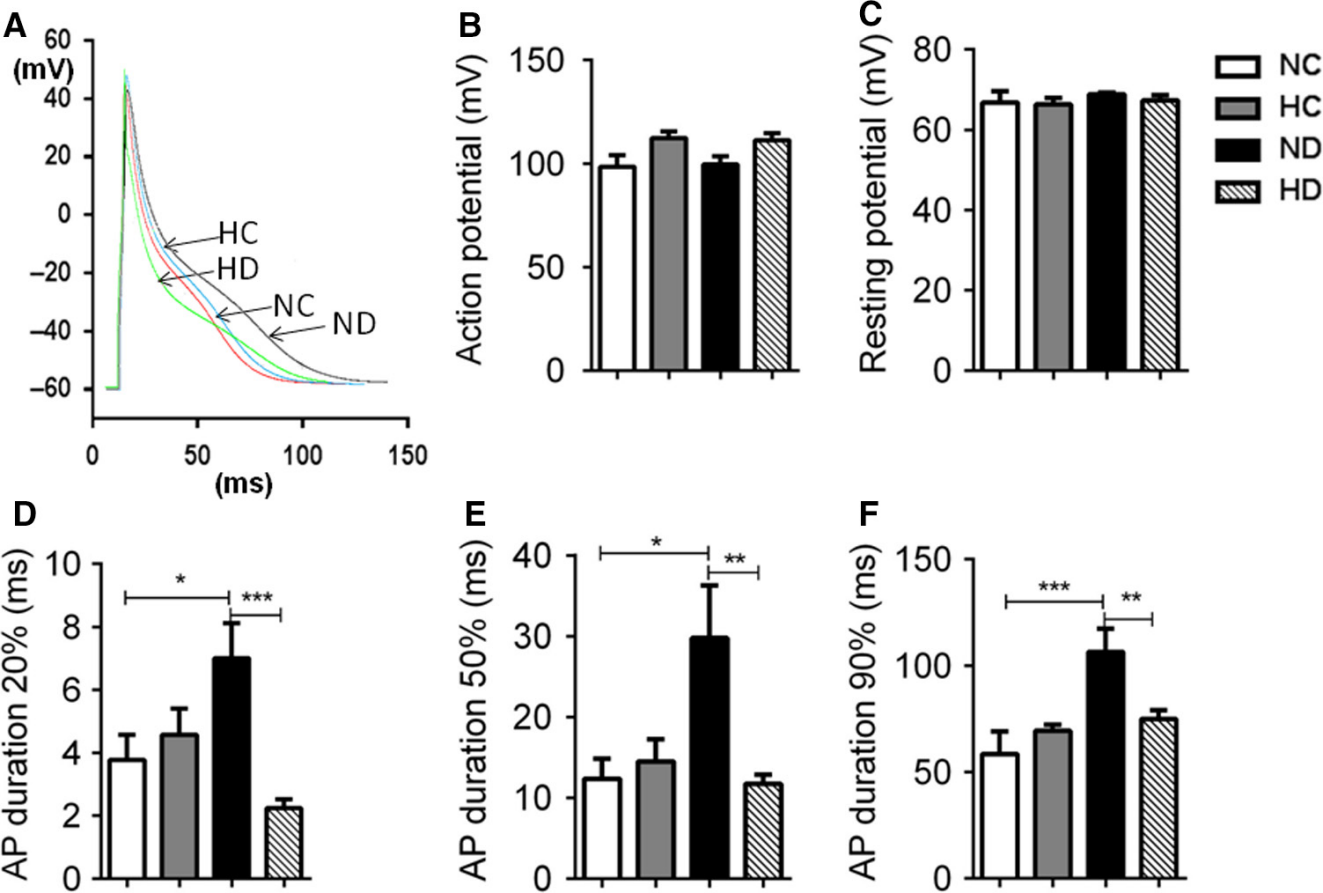
Effect of hexarelin on membrane potential of cardiomyocyte from NC, HC, ND and HD rats. (A) Representative traces of AP from different groups. No changes were found in (B) AP and (C) RMP. Prolonged APD was observed at (D) APD_20_, (E) APD_50_, and (F) APD_90_ in ND cardiomyocytes. HD group showed normalized level of APD at (D) APD_20_, (E) APD_50_, and (F) APD_90_. *n* = 14, 18, 16, 13 cells/6 rats from NC, HC, ND and HD group, respectively. Data were shown as means ± SEM, *P < 0.05, **P < 0.01, ***P < 0.001.

Figure 3 displayed results of RMP, AP and APD from four groups of animals. There was no difference in RMP among different groups of rats. Although an increase in AP amplitude was observed in HC and HD cardiomyocytes, it did not achieved significant. In APD measurement, ND cardiomyocytes showed approximately 1‐fold slower in APD_20_, APD_50_ and APD_90_ compared to NC group but hexarelin treatment made them recovered. No significant difference observed in HC group.

**Figure 4:**
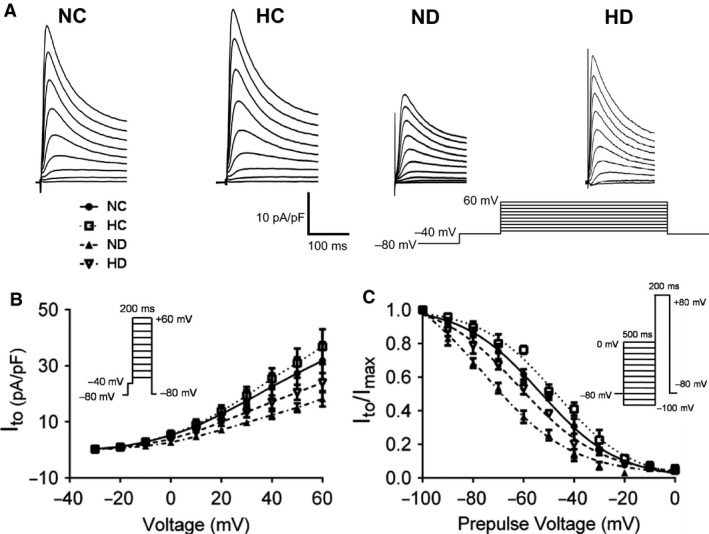
Effect of hexarelin on calcium‐independent transient outward potassium current (*I*_to_) of cardiomyocyte from NC, HC, ND and HD rats. (A) Representative traces of *I*_to_ from different groups. (B) The current–voltage (*I*–*V*) relation and (C) the steady‐state inactivation of *I*_to_. For *I*–*V* relation, *n* = 11, 10, 12 and 13 cells/6 rats from NC, HC, ND and HD group, respectively. For steady‐state inactivation of *I*_to_, *n* = 10, 9, 12, 10 cells/6 rats from NC, HC, ND, and HD group, respectively. Data were shown as means ± SEM.

To further characterize prolonged APD in diabetic cardiomyocytes, we further investigated *I*_to_ which is mainly responsible for APD (Casis et al. 1998; Greenstein et al. 2000). *I*_to_ was measured by applying whole‐cell voltage clamp for current recording. All the other currents were eliminated from the whole‐cell recording through application of blockers and voltage presteps to −40 mV. Representative traces shown in Figure 4 demonstrated that, with increase in testing potential, declined *I*_to_ amplitudes was observed in ND group in comparison with that of cardiomyocytes in NC group. Current–voltage (I–V) relationship analysis of *I*_to_ further proved a decreased current density in STZ‐treated cardiomyocytes, indicating that prolonged APD may be a result of decreased *I*_to_. In addition, the steady‐state inactivation cure of *I*_to_ in ND rat cardiomyocytes was left‐shifted compared to that in control group, reducing availability of *I*_to_ channels. After hexarelin treatment, relatively normalized current density and right‐shifted inactivation curve were displayed in HD group, suggesting hexarelin can restore decreased *I*_to_ and further recover prolonged APD in STZ‐induced diabetic cardiomyocytes.

### Effect of hexarelin on GHS‐R expression of ventricular myocardium from control and diabetic rats

**Figure 5:**
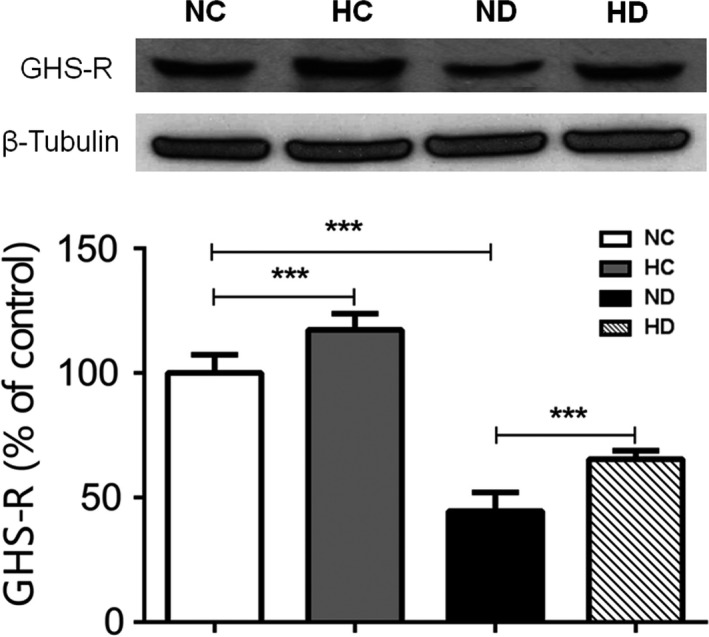
Protein expression of GHS‐R in heart ventricles from NC, HC, ND and HD rats. The level of GHS‐R protein expression was determined by western blot analysis. Rat *β*‐Tubulin was chosen as an internal control. Densitometric quantification of GHS‐R was expressed as percentage relative to control. *n* = 6 from each group. Data were shown as means ± SEM, ***P < 0.001.

The expression of GHS‐R in heart ventricles was examined by western blot measurement. As shown in Figure 5, the level of GHS‐R expression was decreased 50% in STZ group compared to control. As a GHS‐R ligand, hexarelin treatment restored approximately 20% of GHS‐R expression in STZ‐treated ventricular myocardium and also significantly increased that of controls. Therefore, hexarelin was capable of rescuing decreased expression of GHS‐R in STZ‐induced diabetic rat heart and increasing that of normal rat heart, implicating that hexarelin increased expression of GHS‐R may contribute to the beneficial effect of hexarelin on cardiomyocytes.

### Effect of hexarelin on mitochondrial apoptotic signaling of ventricular myocardium from control and diabetic rats

**Figure 6:**
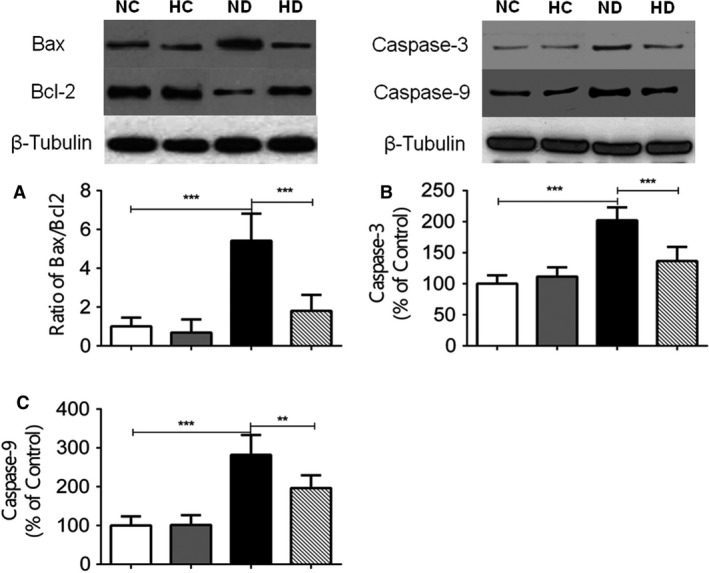
Protein expression of mitochondrial‐mediated apoptotic marker in heart ventricles from NC, HC, ND, and HD rats. The ration of (A) Bax/Bcl‐2, (B) caspase‐3, and (C) caspase‐9 protein expression was determined by western blot analysis. Rat *β*‐Tubulin was chosen as an internal control. Densitometric quantification of caspase‐3 and caspase‐9 was expressed as percentage relative to control. *n* = 6 from each group. Data were shown as means ± SEM, **P < 0.01, ***P < 0.001.

As shown in Figure 6, the ratio of proapoptotic protein, Bax and antiapoptotic protein, Bcl‐2 was significantly increased associated with upregulated apoptosis‐activated cascades, caspase‐3 and caspase‐9 in ND heart ventricles compared to those in NC group. No significant effect of hexarelin administration was observed on these protein expressions in HC group. However, reduced ratio of Bax and Bcl‐2 was found after hexarelin treatment with reduced levels of caspase‐3 and caspase‐9 expression, indicating hexarelin was able to normalize mitochondrial‐related apoptosis (Fig. 6).

## Discussion

In this study, defects of [Ca^2+^]i handling was associated with depressed contractility, prolonged APD and mitochondrial signaling‐initiated apoptosis in STZ‐induced diabetic cardiomyocytes, implicating these alterations contributed to the development of diabetic cardiomyopathy.

Administration of hexarelin recovered the impaired Ca^2+^ homeostasis and the depressed contractility. As a synthetic GHS, the cardio‐protective effect of hexarelin and its natural analog ghrelin has been well characterized in both clinical patients and experimental animals (Bisi et al. 1999b; Broglio et al. 2002; Sun et al. 2010a; Ma et al. 2012a). Clinical studies showed that both hexarelin and ghrelin administration improved cardiac output in healthy volunteers with normal blood pressure and heart rate (Bisi et al. 1999b). Further investigation on patients with coronary artery disease (CAD) and GH deficiency revealed that hexarelin and ghrelin treatment increased left ventricular ejection fraction (LVEF) and cardiac output without alteration of systemic vascular resistance and heart rate (Bisi et al. 1999a; Broglio et al. 2002). These results indicate that GHS is able to improve cardiac contractility in both healthy individuals and patients with onset heart diseases. Single mice ventricular myocyte study in this laboratory exhibited that pre and posttreatment with hexarelin and ghrelin normalized depressed contractility and [Ca^2+^]_i_ transient after *I*/*R* injury through maintaining Ca^2+^ homeostasis by recovering sarcoplasmic reticulum (SR) content, SR Ca^2+^‐ATPase and ryanodine receptor (RyR) activity (Ma et al. 2012a). Another study on isolated adult rat cardiomyocytes from this laboratory also showed that hexarelin and ghrelin exerted a dose‐dependent cardio‐protective effect via GHS‐R as these beneficial effects was abolished after applying inhibitor of GHS‐R (Sun et al. 2010a). Up‐regulation of GHS‐R protein expression was observed with increased cell shortening and [Ca^2+^]_i_ transient of both NC and ND rat hearts in current study, indicating that hexarelin had a positive inotropic effect on rat cardiomyocytes probably through GHS‐R signaling. Moreover, recovered contractility was found in experimental study on STZ‐induced diabetic rat treated by obestatin (coproduct of ghrelin from proghrelin) with restored oxidative balance (Aragno et al. 2012). Insulin treatment has been described to reverse depressed contractility in diabetic rats and rabbits, too (Fein et al. 1981, 1986). Considering our results on decreased blood glucose levels in high fat diet group, it is believed that improved glucose metabolism is partially contributing to normalized contractility and Ca^2+^ homeostasis in STZ‐induced diabetic hearts.

Present study also demonstrated prolonged APD with unchanged RMP and AP in ND cardiomyocytes. APD prolongation in diabetic cardiomyocytes led to suppressed Ca^2+^ extrusion, resulting in increased net Ca^2+^ influx (Shigematsu et al. 1994). Studies on AP of diabetic heart revealed that decreased *I*_to_ was responsible for prolonged APD (Shimoni et al. 1999; Qin et al. 2001). It was stated that depressed *I*_to_ associated with lengthened APD by abolishing influence from other currents (Shigematsu et al. 1994). Furthermore, study on STZ‐induced diabetic heart revealed that, after 14 days of STZ injection, K^+^ channel gene expression was significantly downregulated with decreased *I*_to_ current density (Qin et al. 2001). Impaired glucose metabolism is another contributor to suppressed function of *I*_to_ (Xu et al. 1996a,b; Shimoni et al. 1999; Rozanski and Xu 2002). Voltage‐clamp study demonstrated a recovery of *I*_to_ current density in insulin‐ or metabolic activator, dichloroacetate‐treated diabetic cardiomyocytes (Li et al. 2005). Specifically, STZ‐induced diabetic heart displayed a decrease in thioredoxin reductase with increased thioredoxin activity. Inhibitor of thioredoxin reductase abolished the effect of upregulated *I*_to_ caused by insulin and dichloroacetate. Moreover, normalized *I*_to_ by insulin and dichloroacetate could also be blocked by antagonist of glucose‐6‐phosphate dehydrogenase which produced NADPH required by thioredoxin signaling system. These data indicated that *I*_to_ was regulated in a redox‐sensitive manner by thioredoxin system and decreased thioredoxin reductase activity might lead to suppression of *I*_to_ (Li et al. 2005). Similar study on STZ‐induced diabetic rats also elucidated that increased oxidative stress was associated with diminished *I*_to_ density, resulting in a shift of redox state. Treatment with antioxidant drug restored *I*_to_ density and recovered homeostasis of redox system (Xu et al. 2002), further indicating that oxidative stress was major contributor to depressed *I*_to_.

A growing of evidence has showed that ghrelin acts against oxidative stress by enhancing antioxidant defense (Xu et al. 2007; Tong et al. 2012). It was stated that endogenous ghrelin level was increased during early stage of heart failure development to maintain myocardial energy balance and improve cardiac function as a compensatory self‐protective factor. Ghrelin exerted antioxidative and antiapoptotic effect through improving myocardial mitochondrial function (Xu et al. 2007). In vitro study on hypoxic injury of cardiomyocytes demonstrated that ghrelin administration reduced ROS content with increased Mn‐SOD gene expression and activity via AMPK‐dependent pathway (Tong et al. 2012). Previous study in this laboratory also revealed that both ghrelin and Hex were able to rescue impaired *I*_to_ activity of cardiomyocyte by decreasing MARK‐ROS activity after I/R injury (Ma et al. 2012b). As restored *I*_to_ density by hexarelin was also observed in this study, it may conclude that hexarelin recovers reduced *I*_to_ through improving antioxidant function in STZ‐induced diabetic cardiomyocytes, further normalizes prolonged APD.

Cardiomyocytes are also susceptible to diabetes‐induced cell death. Clinical evidence showed that apoptotic myocytes were 85‐fold higher in diabetic patients than those from control population (Frustaci et al. 2000). Study on STZ‐treated mice displayed an increase in myocardial cell death. Insulin treatment ameliorated myocardial apoptosis with reduced blood glucose level in these mice, suggesting hyperglycemia performed a predominate role in diabetes‐induced myocardial apoptosis (Cai et al. 2002). Mitochondria have been considered as a key regulator of apoptosis with Bcl‐2 family in variety of proapoptotic conditions (Green and Reed 1998; Tsujimoto 1998). It has been exhibited that apoptotic factor, release of cytochrome C, is induced by proapoptotic protein Bax with direct activation of caspases and occurrence of mitochondrial dysfunction such as loss of mitochondrial membrane potential and increase in membrane permeability (Zoratti and Szabo 1995; Liu et al. 1996; Susin et al. 1997; Jurgensmeier et al. 1998; Narita et al. 1998; Shimizu et al. 1998). Bcl‐2, as antiapoptotic molecule, prevents release of cytochrome C and inactivates caspases (Chinnaiyan et al. 1996; Liu et al. 1996; Kluck et al. 1997; Yang et al. 1997). Mitochondrial mediated apoptosis has been well documented in diabetic animal models and patients (Frustaci et al. 2000; Cai et al. 2002; Li et al. 2009; Sun et al. 2014). In vitro study on high glucose‐treated H9c2 cell line demonstrated that increased cardiomyocyte apoptosis was associated with increased intracellular ROS level, leading to elevated activity of caspase‐3 and caspase‐9 with depolarization of mitochondrial transmembrane potential. Furthermore, in vivo investigation on STZ‐induced diabetic mice revealed that up‐regulated Bax and down‐regulated Bcl‐2 was accompanied by an increased release of cytoplasmic cytochrome C (Sun et al. 2014). Similar results were also observed in STZ‐treated rat models (Li et al. 2009). In this report, STZ‐treated rat hearts displayed increased ratio of Bax/Bcl‐2 with increased expression of caspase‐3 and caspase‐9, indicating developed apoptosis via mitochondrial signaling pathways in diabetic cardiomyopathy.

Mitochondrial mediated apoptosis, as stated above, was recovered by hexarelin treatment in current research. In vitro cardiomyocyte study showed that administration of hexarelin decreased angiotensin‐II‐induced apoptosis in H9c2 cell line through the upregulation of Bcl‐2, downregulation of Bax, and inhibition of caspase‐3 activity (Pang et al. 2004), implicating that GHS exerted antiapoptotic effect on cell survival signaling pathways. The results were further proved by in vivo study on rat model with chronic heart failure (Xu et al. 2005). It was demonstrated that hexarelin treatment dramatically decreased apoptotic cell number via recovering imbalanced Bcl‐2/Bax ratio and elevated caspase‐3 expression with increased GHS‐R gene expression. Besides, investigation on H9c2 cardiomyocytes illustrated that high glucose and palmitate‐induced apoptosis could be rescued by ghrelin exposure through increasing anti‐apoptotic expression which further inhibited caspase‐3 and caspased‐9 activity to retain mitochondrial membrane potential and reduce cytoplasmic cytochrome C release. This ghrelin‐induced antiapoptotic effect was associated with activation of PI3K/Akt signaling pathway, indicating GHS may have cell proliferated effect to prevent apoptosis (Kui et al. 2009).

In conclusion, present study illustrated that hexarelin treatment was able to restore STZ‐induced cardiac dysfunction in single myocyte through normalization of depressed contractility, maintenance of impaired Ca^2+^ homeostasis, recovery of prolonged APD and diminished *I*_to_, and inhibition of mitochondrial‐mediated apoptosis with upregulated GHS‐R expression. These results suggest that hexarelin is a potential therapeutic candidate for the treatment of diabetic cardiomyopathy.

## Conflict of Interest

The authors declare that there is no conflict of interest that could be perceived as prejudicing the impartiality of the research reported.

## References

[phy213612-bib-0001] Aragno, M. , R. Mastrocola , C. Ghe , E. Arnoletti , E. Bassino , G. Alloatti , et al. 2012 Obestatin induced recovery of myocardial dysfunction in type 1 diabetic rats: underlying mechanisms. Cardiovasc. Diabetol. 11:129. 23066908 10.1186/1475-2840-11-129 PMC3537569

[phy213612-bib-0002] Bisi, G. , V. Podio , M. R. Valetto , F. Broglio , G. Bertuccio , G. Aimaretti , et al. 1999a Cardiac effects of hexarelin in hypopituitary adults. Eur. J. Pharmacol. 381:31–38. 10528131 10.1016/s0014-2999(99)00537-3

[phy213612-bib-0003] Bisi, G. , V. Podio , M. R. Valetto , F. Broglio , G. Bertuccio , G. Del Rio , et al. 1999b Acute cardiovascular and hormonal effects of GH and hexarelin, a synthetic GH‐releasing peptide, in humans. J. Endocrinol. Invest. 22:266–272. 10342360 10.1007/BF03343555

[phy213612-bib-0004] Bodart, V. , M. Febbraio , A. Demers , N. McNicoll , P. Pohankova , A. Perreault , et al. 2002 CD36 mediates the cardiovascular action of growth hormone‐releasing peptides in the heart. Circ. Res. 90:844–849. 11988484 10.1161/01.res.0000016164.02525.b4

[phy213612-bib-0005] Broglio, F. , F. Guarracino , A. Benso , C. Gottero , F. Prodam , R. Granata , et al. 2002 Effects of acute hexarelin administration on cardiac performance in patients with coronary artery disease during by‐pass surgery. Eur. J. Pharmacol. 448:193–200. 12144941 10.1016/s0014-2999(02)01934-9

[phy213612-bib-0006] Bulgarelli, I. , L. Tamiazzo , E. Bresciani , D. Rapetti , S. Caporali , D. Lattuada , et al. 2009 Desacyl‐ghrelin and synthetic GH‐secretagogues modulate the production of inflammatory cytokines in mouse microglia cells stimulated by beta‐amyloid fibrils. J. Neurosci. Res. 87:2718–2727. 19382238 10.1002/jnr.22088

[phy213612-bib-0007] Cai, L. , W. Li , G. Wang , L. Guo , Y. Jiang , and Y. J. Kang . 2002 Hyperglycemia‐induced apoptosis in mouse myocardium: mitochondrial cytochrome C‐mediated caspase‐3 activation pathway. Diabetes 51:1938–1948. 12031984 10.2337/diabetes.51.6.1938

[phy213612-bib-0008] Casis, O. , M. Iriarte , M. Gallego , and J. A. Sanchez‐Chapula . 1998 Differences in regional distribution of K+ current densities in rat ventricle. Life Sci. 63:391–400. 9714426 10.1016/s0024-3205(98)00287-2

[phy213612-bib-0009] Chinnaiyan, A. M. , K. Orth , K. O'Rourke , H. Duan , G. G. Poirier , and V. M. Dixit . 1996 Molecular ordering of the cell death pathway. Bcl‐2 and Bcl‐xL function upstream of the CED‐3‐like apoptotic proteases. J. Biol. Chem. 271:4573–4576. 8617712 10.1074/jbc.271.9.4573

[phy213612-bib-0010] Choi, K. M. , Y. Zhong , B. D. Hoit , I. L. Grupp , H. Hahn , K. W. Dilly , et al. 2002 Defective intracellular Ca(2+) signaling contributes to cardiomyopathy in Type 1 diabetic rats. Am. J. Physiol. Heart Circ. Physiol. 283:H1398–H1408. 12234790 10.1152/ajpheart.00313.2002

[phy213612-bib-0011] Fein, F. S. , J. E. Strobeck , A. Malhotra , J. Scheuer , and E. H. Sonnenblick . 1981 Reversibility of diabetic cardiomyopathy with insulin in rats. Circ. Res. 49:1251–1261. 7030513 10.1161/01.res.49.6.1251

[phy213612-bib-0012] Fein, F. S. , B. Miller‐Green , B. Zola , and E. H. Sonnenblick . 1986 Reversibility of diabetic cardiomyopathy with insulin in rabbits. Am. J. Physiol. 250:H108–H113. 3510567 10.1152/ajpheart.1986.250.1.H108

[phy213612-bib-0013] Flarsheim, C. E. , I. L. Grupp , and M. A. Matlib . 1996 Mitochondrial dysfunction accompanies diastolic dysfunction in diabetic rat heart. Am. J. Physiol. Heart Circ. Physiol. 271:H192–H202. 10.1152/ajpheart.1996.271.1.H192 8760175

[phy213612-bib-0014] Frustaci, A. , J. Kajstura , C. Chimenti , I. Jakoniuk , A. Leri , A. Maseri , et al. 2000 Myocardial cell death in human diabetes. Circ. Res. 87:1123–1132. 11110769 10.1161/01.res.87.12.1123

[phy213612-bib-0015] Fung, J. N. , I. Seim , D. Wang , A. Obermair , L. K. Chopin , and C. Chen . 2010 Expression and in vitro functions of the ghrelin axis in endometrial cancer. Hormones Cancer 1:245–255. 21761369 10.1007/s12672-010-0047-1 PMC10358077

[phy213612-bib-0016] Green, D. R. , and J. C. Reed . 1998 Mitochondria and apoptosis. Science (New York, NY) 281:1309–1312. 10.1126/science.281.5381.1309 9721092

[phy213612-bib-0017] Greenstein, J. L. , R. Wu , S. Po , G. F. Tomaselli , and R. L. Winslow . 2000 Role of the calcium‐independent transient outward current I(to1) in shaping action potential morphology and duration. Circ. Res. 87:1026–1033. 11090548 10.1161/01.res.87.11.1026

[phy213612-bib-0018] Hayat, S. A. , B. Patel , R. S. Khattar , and R. A. Malik . 2004 Diabetic cardiomyopathy: mechanisms, diagnosis and treatment. Clin. Sci. 107:539–557. 15341511 10.1042/CS20040057

[phy213612-bib-0019] Jurgensmeier, J. M. , Z. Xie , Q. Deveraux , L. Ellerby , D. Bredesen , and J. C. Reed . 1998 Bax directly induces release of cytochrome c from isolated mitochondria. Proc. Natl Acad. Sci. USA 95:4997–5002. 9560217 10.1073/pnas.95.9.4997 PMC20202

[phy213612-bib-0020] Kluck, R. M. , E. Bossy‐Wetzel , D. R. Green , and D. D. Newmeyer . 1997 The release of cytochrome c from mitochondria: a primary site for Bcl‐2 regulation of apoptosis. Science (New York, NY) 275:1132–1136. 10.1126/science.275.5303.1132 9027315

[phy213612-bib-0021] Kojima, M. , H. Hosoda , Y. Date , M. Nakazato , H. Matsuo , and K. Kangawa . 1999 Ghrelin is a growth‐hormone‐releasing acylated peptide from stomach. Nature 402:656–660. 10604470 10.1038/45230

[phy213612-bib-0022] Kui, L. , Z. Weiwei , L. Ling , H. Daikun , Z. Guoming , Z. Linuo , et al. 2009 Ghrelin inhibits apoptosis induced by high glucose and sodium palmitate in adult rat cardiomyocytes through the PI3K‐Akt signaling pathway. Regul. Pept. 155:62–69. 19289146 10.1016/j.regpep.2009.03.003

[phy213612-bib-0023] Li, X. , Z. Xu , S. Li , and G. J. Rozanski . 2005 Redox regulation of Ito remodeling in diabetic rat heart. Am. J. Physiol. Heart Circ. Physiol. 288:H1417–H1424. 15539426 10.1152/ajpheart.00559.2004

[phy213612-bib-0024] Li, C. J. , Q. M. Zhang , M. Z. Li , J. Y. Zhang , P. Yu , and D. M. Yu . 2009 Attenuation of myocardial apoptosis by alpha‐lipoic acid through suppression of mitochondrial oxidative stress to reduce diabetic cardiomyopathy. Chin. Med. J. 122:2580–2586. 19951573

[phy213612-bib-0025] Liu, X. , C. N. Kim , J. Yang , R. Jemmerson , and X. Wang . 1996 Induction of apoptotic program in cell‐free extracts: requirement for dATP and cytochrome c. Cell 86:147–157. 8689682 10.1016/s0092-8674(00)80085-9

[phy213612-bib-0026] Ma, Y. , L. Zhang , J. N. Edwards , B. S. Launikonis , and C. Chen . 2012a Growth hormone secretagogues protect mouse cardiomyocytes from in vitro ischemia/reperfusion injury through regulation of intracellular calcium. PLoS ONE 7:e35265. 22493744 10.1371/journal.pone.0035265 PMC3320867

[phy213612-bib-0027] Ma, Y. , L. Zhang , B. S. Launikonis , and C. Chen . 2012b Growth hormone secretagogues preserve the electrophysiological properties of mouse cardiomyocytes isolated from in vitro ischemia/reperfusion heart. Endocrinology 153:5480–5490. 22948211 10.1210/en.2012-1404

[phy213612-bib-0028] Narita, M. , S. Shimizu , T. Ito , T. Chittenden , R. J. Lutz , H. Matsuda , et al. 1998 Bax interacts with the permeability transition pore to induce permeability transition and cytochrome c release in isolated mitochondria. Proc. Natl Acad. Sci. USA 95:14681–14686. 9843949 10.1073/pnas.95.25.14681 PMC24509

[phy213612-bib-0029] Nobe, S. , M. Aomine , M. Arita , S. Ito , and R. Takaki . 1990 Chronic diabetes‐mellitus prolongs action‐potential duration of rat centricular muscles‐circumstantial evidence for impaired Ca‐2+ channel. Cardiovasc. Res. 24:381–389. 2164883 10.1093/cvr/24.5.381

[phy213612-bib-0030] Pang, J. J. , R. K. Xu , X. B. Xu , J. M. Cao , C. Ni , W. L. Zhu , et al. 2004 Hexarelin protects rat cardiomyocytes from angiotensin II‐induced apoptosis in vitro. Am. J. Physiol. Heart Circ. Physiol. 286:H1063–H1069. 14615277 10.1152/ajpheart.00648.2003

[phy213612-bib-0031] Petrie, M. C. , L. Caruana , C. Berry , and J. J. McMurray . 2002 “Diastolic heart failure” or heart failure caused by subtle left ventricular systolic dysfunction? Heart (British Cardiac Society) 87:29–31. 11751660 10.1136/heart.87.1.29 PMC1766950

[phy213612-bib-0032] Qin, D. Y. , B. Y. Huang , L. L. Deng , H. El‐Adawi , K. Ganguly , J. R. Sowers , et al. 2001 Downregulation of K+ channel genes expression in type I diabetic cardiomyopathy. Biochem. Biophys. Res. Comm. 283:549–553. 11341759 10.1006/bbrc.2001.4825

[phy213612-bib-0033] Rithalia, A. , M. A. Qureshi , F. C. Howarth , and S. M. Harrison . 2004 Effects of halothane on contraction and intracellular calcium in ventricular myocytes from streptozotocin‐induced diabetic rats. Br. J. Anaesth. 92:246–253. 14722178 10.1093/bja/aeh048

[phy213612-bib-0034] Rozanski, G. J. , and Z. Xu . 2002 A metabolic mechanism for cardiac K+ channel remodelling. Clin. Exp. Pharmacol. Physiol. 29:132–137. 11906472 10.1046/j.1440-1681.2002.03618.x

[phy213612-bib-0035] Rubler, S. , Y. Z. Yuceoglu , T. Kumral , A. Grishman , A. W. Branwood , and J. Dlugash . 1972 New type of cardiomyopathy associated with diabetic glomerulosclerosis. Am. J. Cardiol. 30:595–602. 4263660 10.1016/0002-9149(72)90595-4

[phy213612-bib-0036] Shigematsu, S. , T. Maruyama , T. Kiyosue , and M. Arita . 1994 Rate‐dependent prolongation of action‐potential duration in single ventricular myocytes obtained from heartsof rats with streptozotocin‐induced chronic diabetes sustained for 30‐32 weeks. Heart Vessels 9:300–306. 7883652 10.1007/BF01745095

[phy213612-bib-0037] Shimizu, S. , Y. Eguchi , W. Kamiike , Y. Funahashi , A. Mignon , V. Lacronique , et al. 1998 Bcl‐2 prevents apoptotic mitochondrial dysfunction by regulating proton flux. Proc. Natl Acad. Sci. USA 95:1455–1459. 9465036 10.1073/pnas.95.4.1455 PMC19042

[phy213612-bib-0038] Shimoni, Y. , H. S. Ewart , and D. Severson . 1999 Insulin stimulation of rat ventricular K(+) currents depends on the integrity of the cytoskeleton. J. Physiol. 514:735–745. 9882746 10.1111/j.1469-7793.1999.735ad.x PMC2269091

[phy213612-bib-0039] Singh, J. , A. Chonkar , N. Bracken , E. Adeghate , Z. Latt , and M. Hussain . 2006 Effect of streptozotocin‐induced type 1 diabetes mellitus on contraction, calcium transient, and cation contents in the isolated rat heart. Ann. N. Y. Acad. Sci. 1084:178:190. 17151301 10.1196/annals.1372.028

[phy213612-bib-0040] Sun, Q. , Y. Ma , L. Zhang , Y. F. Zhao , W. J. Zang , and C. Chen . 2010a Effects of GH secretagogues on contractility and Ca(2+) homeostasis of isolated adult rat ventricular myocytes. Endocrinology 151:4446–4454. 20610573 10.1210/en.2009-1432

[phy213612-bib-0041] Sun, Q. , W. J. Zang , and C. Chen . 2010b Growth hormone secretagogues reduce transient outward K(+) current via phospholipase C/protein kinase C signaling pathway in rat ventricular myocytes. Endocrinology 151:1228–1235. 20056829 10.1210/en.2009-0877

[phy213612-bib-0042] Sun, X. , R. C. Chen , Z. H. Yang , G. B. Sun , M. Wang , X. J. Ma , et al. 2014 Taxifolin prevents diabetic cardiomyopathy in vivo and in vitro by inhibition of oxidative stress and cell apoptosis. Food Chem. Toxicol. 63:221–232. 24269735 10.1016/j.fct.2013.11.013

[phy213612-bib-0043] Susin, S. A. , N. Zamzami , M. Castedo , E. Daugas , H. G. Wang , S. Geley , et al. 1997 The central executioner of apoptosis: multiple connections between protease activation and mitochondria in Fas/APO‐1/CD95‐ and ceramide‐induced apoptosis. J. Exp. Med. 186:25–37. 9206994 10.1084/jem.186.1.25 PMC2198951

[phy213612-bib-0044] Swynghedauw, B. 1999 Molecular mechanisms of myocardial remodeling. Physiol. Rev. 79:215–262. 9922372 10.1152/physrev.1999.79.1.215

[phy213612-bib-0045] Teshima, Y. , N. Takahashi , T. Saikawa , M. Hara , S. Yasunaga , S. Hidaka , et al. 2000 Diminished expression of sarcoplasmic reticulum Ca(2+)‐ATPase and ryanodine sensitive Ca(2+)Channel mRNA in streptozotocin‐induced diabetic rat heart. J. Mol. Cell. Cardiol. 32:655–664. 10756121 10.1006/jmcc.2000.1107

[phy213612-bib-0046] Tong, X. X. , D. Wu , X. Wang , H. L. Chen , J. X. Chen , X. X. Wang , et al. 2012 Ghrelin protects against cobalt chloride‐induced hypoxic injury in cardiac H9c2 cells by inhibiting oxidative stress and inducing autophagy. Peptides 38:217–227. 23000094 10.1016/j.peptides.2012.06.020

[phy213612-bib-0047] Torsello, A. , E. Bresciani , G. Rossoni , R. Avallone , G. Tulipano , D. Cocchi , et al. 2003 Ghrelin plays a minor role in the physiological control of cardiac function in the rat. Endocrinology 144:1787–1792. 12697684 10.1210/en.2002-221048

[phy213612-bib-0048] Tsujimoto, Y. 1998 Role of Bcl‐2 family proteins in apoptosis: apoptosomes or mitochondria? Genes Cells 3:697–707. 9990505 10.1046/j.1365-2443.1998.00223.x

[phy213612-bib-0049] Westermann, D. , S. Van Linthout , S. Dhayat , N. Dhayat , F. Escher , C. Bucker‐Gartner , et al. 2007 Cardioprotective and anti‐inflammatory effects of interleukin converting enzyme inhibition in experimental diabetic cardiomyopathy. Diabetes 56:1834–1841. 17473225 10.2337/db06-1662

[phy213612-bib-0050] Xu, Z. , K. P. Patel , and G. J. Rozanski . 1996a Intracellular protons inhibit transient outward K+ current in ventricular myocytes from diabetic rats. Am. J. Physiol. 271:H2154–H2161. 8945936 10.1152/ajpheart.1996.271.5.H2154

[phy213612-bib-0051] Xu, Z. , K. P. Patel , and G. J. Rozanski . 1996b Metabolic basis of decreased transient outward K+ current in ventricular myocytes from diabetic rats. Am. J. Physiol. 271:H2190–H2196. 8945940 10.1152/ajpheart.1996.271.5.H2190

[phy213612-bib-0052] Xu, Z. , K. P. Patel , M. F. Lou , and G. J. Rozanski . 2002 Up‐regulation of K(+) channels in diabetic rat ventricular myocytes by insulin and glutathione. Cardiovasc. Res. 53:80–88. 11744015 10.1016/s0008-6363(01)00446-1

[phy213612-bib-0053] Xu, X. B. , J. J. Pang , J. M. Cao , C. Ni , R. K. Xu , X. Z. Peng , et al. 2005 GH‐releasing peptides improve cardiac dysfunction and cachexia and suppress stress‐related hormones and cardiomyocyte apoptosis in rats with heart failure. Am. J. Physiol. Heart Circ. Physiol. 289:H1643–H1651. 15951341 10.1152/ajpheart.01042.2004

[phy213612-bib-0054] Xu, Z. , W. Wu , X. Zhang , and G. Liu . 2007 Endogenous ghrelin increases in adriamycin‐induced heart failure rats. J. Endocrinol. Invest. 30:117–125. 17392601 10.1007/BF03347409

[phy213612-bib-0055] Xu, X. , F. Ding , J. Pang , X. Gao , R.‐K. Xu , W. Hao , et al. 2012 Chronic administration of hexarelin attenuates cardiac fibrosis in the spontaneously hypertensive rat. Am. J. Physiol. Heart Circ. Physiol. 303:H703–H711. 22842067 10.1152/ajpheart.00257.2011

[phy213612-bib-0056] Yang, J. , X. Liu , K. Bhalla , C. N. Kim , A. M. Ibrado , J. Cai , et al. 1997 Prevention of apoptosis by Bcl‐2: release of cytochrome c from mitochondria blocked. Science (New York, NY) 275:1129–1132. 10.1126/science.275.5303.1129 9027314

[phy213612-bib-0057] Yu, J. Z. , B. Rodrigues , and J. H. McNeill . 1997 Intracellular calcium levels are unchanged in the diabetic heart. Cardiovasc. Res. 34:91–98. 9217877 10.1016/s0008-6363(97)00034-5

[phy213612-bib-0058] Zoratti, M. , and I. Szabo . 1995 The mitochondrial permeability transition. Biochem. Biophys. Acta. 1241:139–176. 7640294 10.1016/0304-4157(95)00003-a

